# Social determinants of the impact of hospital management boards on quality management: a study of 109 European hospitals using a parsonian approach

**DOI:** 10.1186/s12913-020-06053-0

**Published:** 2021-01-19

**Authors:** Holger Pfaff, Antje Hammer, Marta Ballester, Kristina Schubin, Michael Swora, Rosa Sunol

**Affiliations:** 1 Institute for Medical Sociology, Health Services Research and Rehabilitation Science, Faculty of Human Science, Faculty of Medicine, University of Cologne, Eupener Strasse 129, 50933 Cologne, Germany; 2 Avedis Donabedian Research Institute (FAD), Universitat Autònoma de Barcelona, Barcelona, Spain; 3 Universitat Autònoma de Barcelona, Barcelona, Spain; 4 Red de Investigación en Servicios de Salud en Enfermedades Crónicas (REDISSEC), Barcelona, Spain

**Keywords:** Quality management, Implementation power, Leadership, Hospital management board, Social capital, Top management team, Consolidated framework for implementation research

## Abstract

**Background:**

The consolidated framework for implementation research states that personal leadership matters in quality management implementation. However, it remains to be answered which characteristics of plural leadership in hospital management boards make them impactful. The present study focuses on social determinants of implementation power of hospital boards using Talcott Parsons’ sociological concept of adaptation, goal attainment, integration, and latency (AGIL), focusing on the G (goal attainment) and I (integration) factors of this concept. The study aims to test the hypothesis that hospitals with management boards that are oriented toward the quality goal (G) and socially integrated (I) (GI boards) are better at implementing quality management than hospitals with boards lacking these characteristics (non-GI boards).

**Methods:**

A cross-sectional mixed-method design was used for data collection in 109 randomly selected hospitals in seven European countries. Data is based on the study “Deepening our understanding of quality improvement in Europe” (DUQUE). We used responses from (a) hospitals’ chief executive officers to measure the variable social integration and the variable quality orientation of the board and (b) responses from quality managers to measure the degree of implementation of the quality management system. We developed the GI index measuring the combination of goal-orientation and integration. A multiple linear regression analysis was performed.

**Results:**

Hospitals with management boards that are quality oriented and socially integrated (GI boards) had significantly higher scores on the quality management system index than hospitals with boards scoring low on these features, when controlled for several context factors.

**Conclusions:**

Our findings suggest that the implementation power of hospital management boards is higher if there is a sense of unity and purpose within the boards. Thus, to improve quality management, it could be worthwhile to increase boards’ social capital and to increase time designated for quality management in board meetings.

## Background

The consolidated framework for implementation research (CFIR) states that personal leadership is an important contextual determinant for the implementation of quality management [1, 2]. However, leadership may be plural [3], as is the case in hospital management boards (HMBs). It remains to be answered which features of plural leadership increase the power of a HMB to implement quality management throughout the hospital.

Research on success characteristics of HMBs provides insights into the importance of the structural characteristics of HMBs. These studies often examine the direct relationship between HMBs and health care quality [4–7], disregarding the mechanisms that transform the political will of plural leaders into positive health care outcomes. The implementation of quality management systems in the hospitals can potentially mediate between HMBs and patient care quality. The CFIR underscores the importance of leadership for implementation. We aim to improve this hypothesis by connecting it with social theory. Frameworks for implementation research, such as CFIR, focus on variables rather than theories; however, we are convinced that we have to use theories to explain how these variables function. Thus, we propose a framework-plus-theory approach in quality research. The CFIR states that leadership commitment is essential for the implementation of innovations and evidence-based practices [2]. We specify this hypothesis using the well-established sociological concept of AGIL by Talcott Parsons [8].

The AGIL scheme [8] assumes that social systems – such as HMBs – have to fulfill four central functions to survive and be productive: adaptation (A), goal attainment (G), integration (I), and latency (L). Because the function “goal attainment” is closely linked to the CFIR notion of leadership commitment, we focus on the factor goal attainment (G factor). However, our assumption is that the HMBs’ plural leadership commitment alone is not sufficient to exert power and influence from the top level (HMB) to the middle and lower levels of hospital management to fully implement a hospital-wide quality management system. Additionally, HMBs need a certain sense of unity and agreement within the top management team. Therefore, based on the AGIL concept, we add the factor social integration (I factor) to the G factor. Social integration makes a group of leaders act like a single leader, making plural leadership a united leadership. The integration factor is advantageous because it allows HMBs to speak with one voice, reach balanced and mature decisions, and communicate the committed decisions via committed board members to the stakeholders they represent (e.g., physicians, nurses, and administrators).

Based on the GI model of system performance [9, 10], we define the GI factor as follows: The GI factor is given in a group if two conditions are met: (1) strong actions of the group to attain specific goals (G factor) and (2) a high degree of social integration within the group (I factor). Top management teams with the GI factor have a sense of unity and purpose, which makes them impactful. Therefore, our hypothesis is that HMBs, which combine high goal attainment with regard to quality (G) and high social integration within the board (I) (GI boards) have greater implementation power than HMBs with low integration and low quality goal orientation (non-GI boards).

## Methods

We used data from the multicenter cross-sectional study “Deepening our understanding of quality improvement in Europe” (DUQuE) [11]. DUQuE fulfilled the ethical requirements for research projects as described in the seventh framework of EU Directorate-General Research. Ethics approval was granted through the Bioethical Committee of the Department of Health of the Government of Catalonia, Spain. Data collection in each country complied with confidentiality requirements according to the national legislation or standards of practice of that country.

Patient records and other information was anonymized and de-identified prior to analysis. The inclusion criteria for hospitals were as follows: (a) having 120 beds or more, (b) delivering care for four medical conditions – acute myocardial infection, stroke, hip fracture, and delivery and (c) having a HMB with at least 2 but no more than 25 board members. The exclusion criterion was missing data in the variables used in the regression analysis. An analysis of the missing data showed only minor differences between the sample obtained after applying only the inclusion criteria (N = 152) and the sample obtained after applying the exclusion criterion as well (N = 109). There were no or only minor differences in the distribution of the following variables: public hospitals (full sample: 82.9% vs. missing-data-free sample: 82.6%), social capital (mean: 3.27 vs. 3.25), quality orientation (mean: 3.86 vs. 3.94), and GI index (GI hospitals: 29.5% vs. 30.3%). There were differences regarding hospital size (hospitals with more than 500 beds: 45.4% vs. 51.3%) and small differences regarding teaching status (40.1% vs. 42.2%). Data collection was performed in 109 randomly selected hospitals in the Czech Republic, France, Germany, Poland, Portugal, Spain, and Turkey. In the present study, we used the responses of the hospitals’ chief executive officers (CEOs) and quality managers obtained through web-based questionnaires. Data were collected between May 2011 and February 2012.

Items used in the questionnaire were either newly developed in English (e.g. quality management system index) or translated from existing measures into English (e.g. social capital scale) or adapted. The final questionnaire has been translated into seven languages following a standard protocol for a forward–backward translation process supervised by local country coordinators [12, 13]. We used a 46-item quality management system index as the dependent variable measuring the degree of implementation of quality management systems in hospitals from the perspective of the quality managers. This index was the sum of scores on the following nine subscales: quality policy documents (three items), quality monitoring by the board (five items), training of professionals (nine items), formal protocols for infection control (five items), formal protocols for medication and patient handling (four items), analyzing performance of care processes (eight items), analyzing performance of professionals (three items), analyzing feedback and patient experiences (three items), and evaluating results (six items). Eight of the nine subscales of this index had sufficient reliability (Cronbach’s alpha: 0.70–0.88), whereas one subscale – analyzing feedback and patient experiences – did not. To ensure content validity, we decided to include this subscale in the quality management system index as well. For details, see Wagner et al. [12]. We used the averaged composite score for the nine subscales ranging from 0 to 27 points.

To measure the first component of the GI index – the G factor – we built the index “hospital board quality goal orientation” as a surrogate variable. Based on the empirical work of Botje and colleagues concerning quality orientation of hospital management boards [14] we measured the goal attainment index by asking the CEOs (a) to assess how frequently quality performance was an item on the executive board’s agenda during the last year (never [1], in a few meetings [2], in most meetings [3], or in every meeting [4]) and (b) to assess what percentage of the hospital (management) board’s meeting time was typically spent on issues of quality performance (10% or less [1], 11–20% [2], 21–30% [3], 31–40% [4], and greater than 40% [5]). This index was built by adding the answers of these two questions. On the basis of this index, we then constructed a dichotomous variable using the tercile split method, creating a binary variable: low quality orientation (low and medium tercile [0]) and high quality orientation (upper tercile [1]). We used the upper tercile split method and not the median split method based on the assumption that it is necessary to have a strong goal orientation in the HMBs to have an impact on the followers across different management levels.

To measure the second component of the GI index – the I factor – we used the scale “social capital within the hospital management board” [15] as a proxy for social integration of the board. It was derived from a validated scale called social capital of healthcare organizations from employees’ perspective (SOCAPO-E), which has been used in previous studies [16, 17]. For this study, the six items of this scale were adapted in order to measure social capital among hospital management board members (SOCAPO-B). The scale consists of six items (Cronbach’s alpha: 0.91) measuring the amount of unity and agreement, common values, mutual trust, mutual helpfulness, “we” feeling, and good social climate in the HMBs. The scale reflects central features of the communal aspects of social capital [17–19]. We used a 4-point Likert scale from “strongly disagree” (1) to “strongly agree” (4). To construct the dichotomous variable “integration index”, which separates HMBs scoring high in social capital (1) from those scoring low (0). Again, we used the upper tercile split and not the median split method based on the assumption that for having adequate implementation power, a quite high amount of unity and cohesion within HMBs is needed.

Based on the goal–integration model [9; 10] we developed a **GI index** by adding both dichotomous variables, resulting in an index with three values: 0 (HBM with low quality goal orientation and low social capital), 1 (HBM low in one of these two variables), and 2 (HBM high in both variables). Thus, we did not differentiate between the other two combinations – HMB with high quality goal orientation but without high social capital and HMB without high quality goal orientation but with high social capital – because both combinations are considered equivalent in promoting collective action. We used board size, ownership, hospital size, teaching status of hospital, and country as control variables. For the multivariate analysis, we used multiple linear regression with a restricted and a full model using IBM SPSS STATISTICS version 26.

## Results

### Sample characteristics and descriptive statistics

The dataset included in this analysis contained only hospitals with complete data on all variables used in the multiple regression model (N = 109). The sample included seven countries (see Table 1); 82.6% were public hospitals, and 42.2% were teaching hospitals. Approximately 11% of the hospitals had less than 200 beds; 37.6% had 200–500 beds; 33.9% had 501–1000 beds; and 17.4% had more than 1000 beds. The average number of HMB members in our sample was 7.6 (SD = 4.1). The mean of the social capital scale – as one component of the GI index – was 3.3 (SD = 0.61), measured on a scale ranging from 1 to 4. The dichotomization of this scale using an upper tercile split showed that 64.2% and 35.8% of the hospitals, respectively, had low and high HMB social capital. The mean of the hospital board quality goal orientation index – the other component of the GI index – was 3.9 (SD = 1.61), measured on a scale ranging from 0 to 7. The dichotomization of this scale using an upper tercile split indicated that 58.7% and 41.3% of the hospitals, respectively, showed low and high HMB quality goal orientation. The creation of the goal–integration index led to three GI levels, with 40.4% of the HMBs (N = 44) on the lowest level, 42.2% (N = 46) on the middle level, and 17.4% (N = 19) on the highest level (see Table 2). Moreover, the average mean of the outcome variable quality management system index was 19.3 (SD = 4.6) on a scale ranging from 0 to 27.

**Table 1:** Percentage of the hospitals in European countries participating in DUQUE used in the analysis (N = 109)

		N	%
**Country**	Czech Republic	19	17.4
	France	17	15.6
	Germany	7	6.4
	Poland	17	15.6
	Portugal	19	17.4
	Spain	19	17.4
	Turkey	11	10.1
	Total	109	100.0

**Table 2:** Goal–integration (GI) index (*N* = 109)

Characteristic of Hospital Management Board (HMB)	N	%
Quality goal orientation of HMB (G index)		
0 – HMB low in quality goal orientation	64	58.7
1 – HMB high in quality goal orientation	45	41.3
Total	109	100
Social capital of HMB (I index)		
0 – HMB low in social capital	70	64.2
1 – HMB high in social capital	39	35.8
Total	109	100
GI index		
0 – HMB low in social capital and quality goal orientation	44	40.4
1 – HMB low in social capital OR quality goal orientation	46	42.2
2 – HMB high in social capital and quality goal orientation	19	17.4
Total	109	100

### Regression analyses

Checking for outliers with Cook’s distance proved to be negative, indicating no outliers. The results of the linear regression analysis (see Table 3) indicate a significant association between the boards’ GI index and the quality management system index. Compared with the restricted model containing the control variables (explained variance: 27,6%; *p* < 0.001), the introduction of the GI factor into the equation led to a significant increase of 5,7% in explained variance (*p* < 0.05); the full model explained 33% of the variance in the quality management system index. Hospitals with boards high in the GI index had significantly better scores on the quality management system index than hospitals with boards low in quality goal orientation and in social capital (reference group: non-GI-board hospitals; *p* < 0.01). The results also showed that hospitals that meet only one of both impact requirements – HMB quality goal orientation or HMB social capital – have an advantage over hospitals that fulfill none of the two requirements, but this advantage was not significant. We conducted regression diagnostics by checking the assumptions of linearity, variance homogeneity and normally distributed errors by means of scatter- and QQ-plots of the standardized residuals, which proved to be well-behaved. Furthermore, there were no signs of multicollinearity as measured by the condition index.

**Table 3:** Regression analysis with quality management system index as dependent variable (*N* = 109)

	Regression coefficient	*p*-value		CI (95%)	Regression coefficient	*p*-value	CI (95%)
	**Step 1: Restricted model**				**Step 2: Full model**		
(Constant)	20.8		**0.000**	16.3; 25.2	20.6	**0.000**	16.3; 25.0
**Control variables**							
Czech Republic (1)	1.7		0.38	-2.1; 5.6	0.7	0.70	-3.1; 4.5
France (1)	-1.1		0.61	-5.5; 3.2	-2.0	0.36	-6.3; 2.3
Poland (1)	2.6		0.33	-2.6; 7.7	1.7	0.52	-3.5; 6.8
Portugal (1)	-1.0		0.63	-5.2; 3.1	-1.9	0.37	-6.0; 2.2
Spain (1)	-2.3		0.34	-7.1; 2.5	-3.3	0.16	-8.1; 1.4
Turkey (1)	4.2		0.08	-0.5; 8.9	2.3	0.35	-2.5; 7.1
Number of board members	-0,03		0.84	-0.3; 0.3	-0.014	0.92	-0.3; 0.3
Number of beds between 200 and 500 (2)	-1.0		0.54	-4.2; 2.2	-2.2	0.19	-5.4; 1.1
Number of beds between 501 and 1000 (2)	-0.5		0.79	-3.9; 3.0	-1.5	0.40	-4.9; 2.0
Number of beds more than 1000 (2)	-0.8		0.69	-4.7; 3.1	-1.5	0.44	-5.4; 2.4
Teaching Hospital	-1.8		0.29	-5.3; 1.6	-1.8	0.29	-5.2; 1.5
Public Hospital	-0.2		0.88	-3.0; 2.6	0.6	0.68	-2.2; 3.3
**GI Index**							
GI index: middle (3)					1.6	0.08	-0.2; 3.4
GI index: high (3)					3.3	**0.007**	0.9; 5.7
**ΔR**^**2**^	**0.276**		**0.001**		**0.057**	**0.022**	
**R**^**2**^	**0.276**		**0.001**		**0.333**	**0.000**	

(1) Dummy variable: reference category: Germany

(2) Dummy variable: reference category: number of beds lower than 200

(3) Dummy variable: reference category: GI index = low

## Discussion

Implementation of quality management systems in hospitals is one of the central tasks in quality and safety. According to the CFIR, one way to promote this is using leadership commitment [2]. In the case of HMBs, leadership is plural leadership. The present study aimed to investigate which conditions foster the implementation power of plural leadership. To answer this question, we considered the HMB as a social system and used Parsons’ social system theory.

On the basis of Parsons’ sociological concept of AGIL, we proposed that the combination of goal attainment (G) and social integration (I) – the GI factor – is a social precondition for any collective endeavor such as the implementation of a hospital-wide quality management system. In general, our empirical results support the hypothesis that, in European hospitals, quality management strategies are fully implemented more often if the hospitals are managed by hospital boards with a high GI factor. After adjusting for various potential confounding variables, hospitals with GI boards showed a higher score on the quality management system index than hospitals led by non-GI boards. Hypothetically, this means that hospitals could realize an absolute improvement of 3.3 points on the 27-point quality management system index (11% improvement) if there is strong emphasis on quality performance and a sense of unity within the HMBs. There was no significant increase compared with the reference group (low GI-board hospital) if the HMB either had quality orientation only (G) or a sense of unity only (I). Therefore, having a board with only one of these success factors does not seem sufficient. The possible explanations for this finding need to be explored.

Our results correspond with the upper echelons theory [20]. This theory states that certain characteristics of top management teams influence the decision making of the top team itself as well as the decisions and behaviors of others outside the team, especially employees in the organization [20].

Thus, we have two types of HMB impact: (a) an inward impact and (b) an outward impact. One inward impact is that HMBs with a high degree of social integration can make quick collective decisions owing to shared values and a common will to reach agreement [21]. The other inward impact is that socially integrated HMBs are able to integrate different cultures and stakeholders within the HMB through group-bonding processes, leading to more culturally balanced decisions and less social conflicts within HMBs. Shared leadership studies support this perspective [22, 23].

An important outward impact of an integrated and committed HMB is its power to influence the lower hierarchical levels and its power to implement innovations on these levels. This implementation power depends on the formal power of the HMB to make decisions and to lead the hospital. Moreover, this implementation power stems from the social contagion effect of committed groups. Social contagion describes the diffusion of emotions, cognitions, attitudes, or behaviors in groups, networks, and organizations [24]. Studies showed that leaders’ mood and passion [25] as well as their commitment towards a certain goal (e.g., quality goal) are contagious to the followers [26, 27]. Furthermore, the implementation power is based on the unity of the group. This unity bundles together the individual energies of the board members forming a cohesive top management team with high social energy that can be used to influence people (I: integration). Additionally, the implementation power stems from the role model function of a quality-oriented HMB. Quality-striving boards are an inspiring example for attaining quality goals throughout the hospital on all hierarchical levels (G: goal attainment). The Ohio model of leadership further supports this informal power perspective [28]. The Ohio model states that successful leadership combines two dimensions: (a) consideration (fostering the quality of the social relationships between leaders and followers) and (b) initiating structure (the degree to which a leader is oriented toward goal attainment) [29]. The first dimension relates to the I factor in the AGIL concept, and the second dimension relates to the G factor.

Implementation power is important for transferring quality practices to all hospital levels because the implementation of quality-oriented structures and processes affects employees with vested interests and involves activating resources controlled by other managers [30]. Empirical results suggest that “in order to have effective strategy implementation processes (…) top management teams first need to minimize within-team fragmentation pressures to achieve integration” [31]. Therefore, strengthening the social capital of HMBs by improving the team climate is important to foster the implementation power of the board.

### Strengths and limitations

The presented data are based on a cross-national, mixed-method study in seven European countries. We mostly used validated scales. Moreover, we used different sources of data for the GI index (CEO questionnaire) and for the quality management system index (quality manager questionnaire) to diminish the risk of common method variance bias [32]. However, the present analyses have certain limitations that are worth mentioning. First, the study is based on a cross-sectional design and does not allow us to draw causal conclusions. Future studies should strive for longitudinal designs. Second, social capital, quality goal orientation, and quality management system are self-reported measures based on the questionnaires answered by the CEOs and quality managers. These variables are not based on objective measurements (e.g. observations), which would be an additional goal for future studies. Therefore, they represent the perception of these professionals, which should be considered when interpreting the results. Third, the two-item index “hospital management board quality goal orientation” is not a validated measure yet. However, this index is based on quality management research and has been developed and used in a previous study as a reliable, fact-based measure [14]. By dichotomizing this index, we additionally facilitated the robustness of the measure. Fourth, one could criticize the key informant approach used; we chose this approach owing to its practicability. Nevertheless, the use of key informants is common practice in business research and health services research. Fifth, despite high response rates, our results are restricted to 109 hospitals because of missing values in the quality management system index. Further analysis showed no significant differences between the original sample and the analyzed sample of 109 hospitals. Sixth, we considered most potential confounders, but uncontrolled confounding cannot be completely eliminated. Seventh, despite our efforts to use a validated approach for backward-forward translation [13], we cannot fully rule out the possibility that the meaning of the items used might differ across national cultures and regulatory environments.

## Conclusions

To the best of our knowledge, this is the first large-scale study that explored two possible social determinants of the impact of hospital boards on quality management: quality goal orientation (G factor) and social integration (I factor). Due to the limitations of this study, we have to be careful with conclusions. However, we can state that this study could be regarded as a hypothesis generating study. Hence, the present study improves the consolidated framework for implementation research by generating the hypothesis that shared commitment towards quality performance combined with social integration within HMBs could possibly increase the power of the HMBs to implement quality management systems within the hospital. Regarding future research, longitudinal studies are required to further examine the relationship between HMB characteristics and implementation of quality management systems. Regarding quality management practices, results indicate tentatively that two practical measures could strengthen the implementation of evidence-based quality in hospitals: (1) fostering the social capital in HMBs using team development and (2) reserving time to discuss quality on the agenda of the board meetings.

## Data Availability

The datasets used and/or analyzed during the present study are available upon reasonable request. Please send your request to Rosa Sunol (rsunol@fadq.org; ORCID: 0000-0003-1902-833X).

## Abbreviations

CFIR: Consolidated Framework for Implementation Research
GI: Goal–Integration
DUQUE: Deepening our Understanding of Quality improvement in Europe
CEOs: Chief Executive Officers
HMBs: Hospital Management Boards
AGIL: Adaptation, Goal attainment, Integration, Latency
SD: Standard Deviation
CI: Confidence Interval
